# GVMD-NLM: A Hybrid Denoising Method for GNSS Buoy Elevation Time Series Using Optimized VMD and Non-Local Means Filtering

**DOI:** 10.3390/s26020522

**Published:** 2026-01-13

**Authors:** Huanghuang Zhang, Shengping Wang, Chao Dong, Guangyu Xu, Xiaobo Cai

**Affiliations:** 1 School of Surveying and Geoinformation Engineering, East China University of Technology, Nanchang 330013, China; 2023120492@ecut.edu.cn (H.Z.); gyxu@ecut.edu.cn (G.X.); caixb@ecut.edu.cn (X.C.); 2 Key Laboratory of Marine Environment Survey Technology and Application, Ministry of Natural Resources, Guangzhou 510300, China; 3 South China Sea Marine Survey Center, Ministry of Natural Resources, Guangzhou 510300, China

**Keywords:** GNSS elevation time series, grey wolf optimizer, variational mode decomposition, non-local means, signal denoising

## Abstract

**Highlights:**

**What are the main findings?**

- An improved Grey Wolf Optimizer was proposed to automatically and optimally determine the key parameters for VMD, overcoming the reliance on empirical presetting and enhancing its performance for GNSS signals.
- A hybrid denoising strategy was developed that combines the optimized VMD with Non-Local Means filtering, using Sample Entropy to effectively separate and process noise and signal components for superior denoising.

**What are the implications of the main findings?**

- The method provides an objective and automated solution for parameter selection and noise-signal separation in GNSS buoy data processing, improving its reliability.
- This approach achieves high-quality denoising for GNSS buoy elevation time series in coastal waterways, offering a more accurate data foundation for applications in waterway hydrodynamics and water level monitoring and research.

**Abstract:**

GNSS buoys are essential for real-time elevation monitoring in coastal waterways, yet the vertical coordinate time series are frequently contaminated by complex non-stationary noise, and existing denoising methods often rely on empirical parameter settings that compromise reliability. This paper proposes GVMD-NLM, a hybrid denoising framework optimized by an improved Grey Wolf Optimizer (GWO). The method introduces an adaptive convergence factor decay function derived from the Sigmoid function to automatically determine the optimal parameters (*K* and α) for Variational Mode Decomposition (VMD). Sample Entropy (SE) is then employed to identify low-frequency effective signals, while the remaining high-frequency noise components are processed via Non-Local Means (NLM) filtering to recover residual information while suppressing stochastic disturbances. Experimental results from two datasets at the Dongguan Waterway Wharf demonstrate that GVMD-NLM consistently outperforms SSA, CEEMDAN, VMD, and GWO-VMD. In Dataset One, GVMD-NLM reduced the RMSE by 26.04% (vs. SSA), 17.87% (vs. CEEMDAN), 24.28% (vs. VMD), and 13.47% (vs. GWO-VMD), with corresponding SNR improvements of 11.13%, 7.00%, 10.18%, and 5.05%. In Dataset Two, the method achieved RMSE reductions of 28.87% (vs. SSA), 17.12% (vs. CEEMDAN), 18.45% (vs. VMD), and 10.26% (vs. GWO-VMD), with SNR improvements of 10.48%, 5.52%, 6.02%, and 3.11%, respectively. The denoised signal maintains high fidelity, with correlation coefficients (*R*) reaching 0.9798. This approach provides an objective and automated solution for GNSS data denoising, offering a more accurate data foundation for waterway hydrodynamics research and water level monitoring.

## 1. Introduction

The Global Navigation Satellite System (GNSS) is a cornerstone of modern Earth science observation infrastructures. Capitalizing on its high precision and real-time capabilities, GNSS supplies high-quality observational data for scientific research [1]. In the context of coastal waterway monitoring, buoys are indispensable, and GNSS buoys, in particular, serve as vital technological platforms. They facilitate real-time measurement of elevation coordinates in coastal waterway environments [2].

In practice, GNSS elevation coordinate time series are often contaminated by various disturbances and noise [3]. In the broader field of remote sensing and environmental monitoring, recent studies have demonstrated that employing intelligent data processing frameworks and multi-source fusion strategies is essential for ensuring signal robustness in complex noise-prone environments, as evidenced in applications ranging from precision agriculture [4] to geospatial analysis. Current research efforts are predominantly directed toward noise processing for terrestrial GNSS coordinate time series, whereas denoising studies specific to GNSS elevation time series in coastal waterway environments remain comparatively limited [5]. Consequently, investigating denoising techniques for GNSS buoy elevation coordinate time series is of considerable significance. It is worth noting that although GNSS provides three-dimensional coordinates (North, East, and Up), this study focuses exclusively on the elevation component. This selection is driven by two main factors: First, from a hydrodynamic perspective, the vertical motion of the buoy directly reflects key hydrological parameters such as tides and waves, whereas horizontal motion is largely governed by low-frequency anchor drift, which is not the primary focus of wave monitoring. Second, due to satellite geometry and atmospheric delays, the vertical component of GNSS typically exhibits a lower signal-to-noise ratio compared to horizontal components. Therefore, verifying the denoising effectiveness on the elevation time series presents a more challenging and representative test for the proposed algorithm.

Common denoising methods for GNSS elevation coordinate time series include wavelet denoising (WD) [6,7,8], empirical mode decomposition (EMD) [9,10], and singular spectrum analysis (SSA) [11,12]. For instance, a method combining a Hausdorff distance screening criterion with wavelet decomposition was proposed in [13] to effectively identify and eliminate noise. However, WD lacks robust theoretical guidance for selecting the optimal basis function, and prevailing threshold determination methods often rely on specific assumptions, exhibiting limited adaptability. The study in [14] applied EMD to correct periodic terms in GNSS time series from continuous stations, offering insights and a theoretical basis for kinematic GNSS observations. Nonetheless, EMD is susceptible to mode mixing and significant end effects, resulting in unreliable IMFs at the data boundaries and compromising the decomposition’s reliability. To mitigate EMD’s drawbacks, Refs. [15,16] introduced an EMD filtering method based on a continuous mean square error criterion and a complementary ensemble EMD (CEEMD) algorithm, respectively. However, the approach in [15] involves subjectivity in identifying the boundary component based on the first significant change in the screening criterion. The method in [15] suffers from several key limitations: pronounced sensitivity to data gaps, potential distortion of IMFs, and the absence of a framework for quantifying uncertainty in individual IMF components. An Adaptive Singular Spectrum Analysis method was proposed in [17], which adaptively determines the embedding window size by integrating spectrum analysis with an improved trace matrix and automatically selects principal components for reconstruction, thereby enabling efficient and accurate extraction of time-varying periodic signals from GNSS height time series. While this method demonstrates markedly superior computational efficiency over traditional techniques, its strategy of using spectrum analysis to guide window size selection still has constraints. VMD, introduced by Dragomiretskiy et al. [18], is a signal processing technique resistant to mode mixing. It can effectively separate IMF components through frequency-domain partitioning and exhibits excellent noise robustness, leading to its widespread application in signal denoising. However, VMD requires predefining the number of IMF components (*K*) and the quadratic penalty factor (*α*). Research indicates that the value of *α* considerably influences the decomposition outcomes [19,20]. In practice, both *K* and *α* are often set manually. Given the complexity and variability of real-world signals, relying solely on empirical parameter presets makes it difficult to achieve an ideal decomposition [21].

To address the limitations of empirical parameter settings and signal over-smoothing, this paper proposes the GVMD-NLM framework. Unlike existing GWO-VMD variants that often rely on linear decay strategies risking premature convergence, our method introduces a nonlinear Sigmoid-based convergence mechanism to robustly optimize the VMD parameters (*K* and *α*). Furthermore, distinct from standard hybrids that may indiscriminately suppress high-frequency components, we integrate Non-Local Means (NLM) filtering to strictly target noise components identified by Sample Entropy. This approach delineates a clear novelty boundary by resolving the critical trade-off between automated denoising efficiency and the preservation of hydrodynamic structural details. Consequently, the main contributions of this work are summarized as follows:

Methodological Innovation: We propose an improved GWO algorithm incorporating a “slow-fast-slow” nonlinear decay mechanism based on the Sigmoid function. This strategy effectively overcomes the limitations of linear decay found in traditional optimizers, ensuring global convergence and precise parameter selection for VMD without manual intervention.

Strategic Integration: We develop a distinct GVMD-NLM framework that resolves the trade-off between denoising strength and detail retention. By utilizing Sample Entropy to strictly segregate noise-dominant modes and applying NLM filtering only where necessary, the method recovers subtle hydrodynamic information that is typically lost in conventional low-pass filtering approaches.

Application Validity: The proposed method provides a validated solution for GNSS buoy elevation time series in dynamic coastal waterways. Experimental evidence confirms it significantly outperforms state-of-the-art baselines by reducing the RMSE by over 13% and maintaining high spectral consistency with physical water level variations.

## 2. Principles and Methods

### 2.1. Improved Grey Wolf Optimizer Algorithm

The classical grey wolf optimizer exhibits limitations when handling bivariate collaborative optimization tasks, primarily because its preset convergence coefficient decay mechanism is unsuitable for the demands of high-dimensional space search [22,23]. To address this shortcoming, a grey wolf optimization algorithm incorporating a dynamic adjustment mechanism is designed. In the standard GWO framework, the adjustment coefficient A directly influences the dynamic characteristics of the wolf pack’s encircling behavior, and its evolution is governed by the core parameter *a*. Therefore, the optimal selection of *a* will significantly impact the algorithm’s exploitation and exploration capabilities in complex optimization scenarios. When *a* > 1, the wolf pack’s search step size exhibits an expansion trend, which aids in enhancing the algorithm’s global exploration performance and effectively avoids premature convergence. Conversely, when *a* < 1, the search step size shows a contraction characteristic, prompting the algorithm to focus on the neighborhood of the optimal solution for fine-grained search. In this case, local exploitation capability is significantly improved, and convergence speed accelerates. However, existing studies predominantly employ a linear decay paradigm to control the evolution of parameter *a*. This single adjustment mode struggles to address the multi-peak characteristics inherent in dual-parameter optimization challenges. To address this, we constructed an improved adaptive decay function derived from the Sigmoid function. The derivation process is structured as follows:

Prototype Selection: The standard Sigmoid function, defined as Equation (1), is selected as the mathematical prototype due to its smooth, nonlinear “S-shaped” transition characteristics and its value range of (0, 1).

(1) $$S(x)=\frac{1}{1+e^{-x}}$$

Target Value Mapping: The convergence factor *a* in GWO requires a decay trajectory from 2 to 0. To map the Sigmoid output to this target range, we first invert the trend using 1−S(x) (transforming the range to 1→0) and then scale it by a factor of 2. The preliminary form is expressed as Equation (2).

(2) $$\alpha =2\times (1-\frac{1}{1+e^{-x}})$$

Domain Transformation: The input variable must be transformed from the unbounded domain *x* to the specific iteration domain $N_{i}\in [0,N_{\max}]$. To ensure the nonlinear inflection point aligns with the middle of the iterative process, we normalize $N_{i}$ and center it using Equation (3).

(3) $$\frac{N_{i}}{N_{\max}}-0.5$$

Slope Modulation: To control the steepness of the decay curve (i.e., the rate of convergence), a modulation factor μ is introduced. Through empirical sensitivity analysis, we set μ=15. This specific value creates a “slow-fast-slow” decay pattern, maintaining a broad search capability for the first half of iterations before rapidly converging for fine-grained exploitation.

Integrating these four steps, the final adaptive convergence factor is formulated as Equation (4):

(4) $$\alpha =\frac{2}{1+e^{15(N_{i}-N_{z}/2)/N_{z}}}$$

where $N_{i}$ is the current iteration number, and $N_{z}$ is the total number of iterations for the algorithm.

The following describes the principles of the improved Grey Wolf Optimizer algorithm:

(1) Social Hierarchy Modeling

In the GWO algorithm, the social hierarchy is mathematically modeled to represent the fitness ranking of candidate solutions. The grey wolf population can be classified into φ, *β*, *δ*, and *ω* wolves. The φ wolf is the individual with the best fitness, representing the current optimal solution found by the algorithm. The *β* wolf is the individual with the second-best fitness, assisting the φ wolf in exploration. The *δ* wolf is the individual with the third-best fitness. The *ω* wolves comprise the remaining individuals, which update their positions by following the top three [24,25].

(2) Encircling Prey

This phase simulates the behavior of the pack encircling prey to progressively narrow down the potential search area. During the optimization process, the mathematical model of this encircling behavior defines the boundaries of the search space around the current best solution, given by Equation (5):

(5) $$\left\{\begin{array}{l} D=\left|C\cdot X_{p}(t)-X(t)\right|\\ X(t+1)=X_{p}(t)-A\cdot D \end{array}\right.$$

where t denotes the current iteration number; the symbol · represents multiplication; *D* is the distance between the grey wolf and the prey; $X_{p}(t)$ indicates the prey position (current optimal solution); X(t) signifies the current position of the grey wolf; **A** and **C** are coefficient vectors, defined as $A=2ar_{1}-a$, $C=2\cdot r_{2}$, with *a* adaptively decreasing from 2 to 0 during iterations; $r_{1}$ and $r_{2}$ are random vectors within the range [0, 1]. By appropriately adjusting the values of parameters **A** and **C**, the algorithm can effectively search for other surrounding points in the vicinity of the optimal solution.

(3) Hunting Prey

The hunting phase corresponds to the position update mechanism, where the search agents (ω wolves) adjust their positions based on the spatial information of the top three solutions. The φ,β and δ wolves guide the pack to update positions towards the global optimum, as shown in Equations (6)–(8):

(6) $$\left\{\begin{array}{l} D_{\varphi }=|C_{1}\cdot X_{\varphi }-X|\\ D_{\beta }=|C_{2}\cdot X_{\beta }-X|\\ D_{\delta }=|C_{3}\cdot X_{\delta }-X| \end{array}\right.$$

(7) $$\left\{\begin{array}{l} X_{1}=X_{\varphi }-A_{1}\cdot D_{\varphi }\\ X_{2}=X_{\beta }-A_{2}\cdot D_{\beta }\\ X_{3}=X_{\delta }-A_{3}\cdot D_{\delta } \end{array}\right.$$

(8) $$X(t+1)=\frac{X_{1}+X_{2}+X_{3}}{3}$$

Here, Equations (6) and (7) define the step size and direction of omega wolves moving towards the φ,β and δ wolves; the latter equation determines the location of the prey.

(4) Attacking Prey

Attacking Prey This stage represents the final convergence of the algorithm, where the search agents focus on exploiting the identified optimal region. This behavioral transition is governed by the linear decrease in the convergence factor *a* from 2 to 0. When *|A|* < 1, the algorithm initiates the local exploitation phase; the value of *A* diminishes, and the wolf pack tightens its encirclement to conduct a fine-grained search. Conversely, when *|A|* > 1, the algorithm demonstrates global exploration behavior, guiding the pack to progressively converge toward the optimal solution through successive iterations.

To rigorously verify the effectiveness of the proposed nonlinear convergence strategy, a comparative ablation study was conducted under identical computational budgets. We evaluated three variants: the Original GWO (which utilizes a linear decay strategy), a GWO variant with Exponential Decay, and the proposed Sigmoid-GWO. All algorithms were configured with a population size of 30 and a maximum of 20 iterations, with results averaged over 50 independent runs to mitigate stochastic randomness.

**Figure 1:**
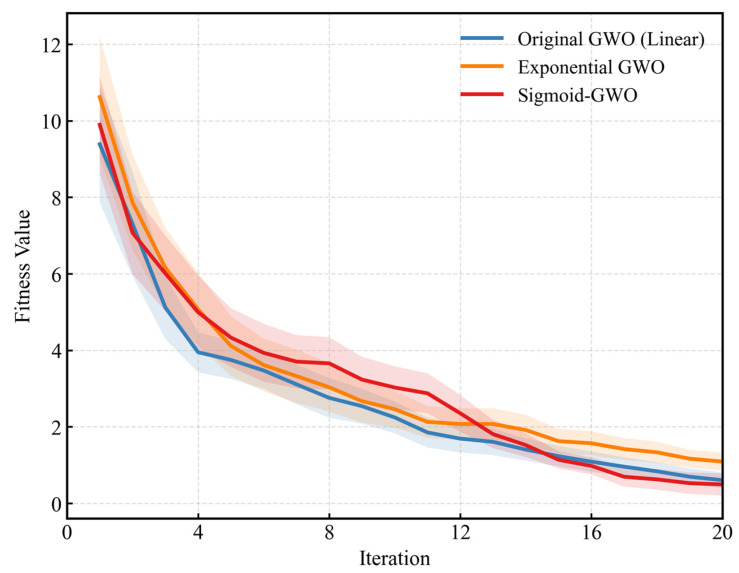
Convergence trajectories of different decay strategies under equal computational budgets.

Figure 1 illustrates the convergence trajectories of these strategies. A distinct behavioral pattern confirms the theoretical advantage of the proposed method. As observed in the initial phase (iterations 1–10), the proposed Sigmoid-GWO maintains a relatively higher fitness value compared to the Original GWO. This deliberate “slow decay” mechanism effectively preserves population diversity, preventing the algorithm from prematurely converging to local optima during the global exploration phase. Subsequently, around the 12th iteration, the Sigmoid-GWO curve accelerates its descent, crossing below the Vanilla GWO curve. This transition demonstrates a swift and effective shift from exploration to precise local exploitation.

The statistical results presented in Table 1 quantitatively confirm this superiority. The proposed Sigmoid-GWO achieved the lowest mean fitness value of 0.4928. This represents an improvement of approximately 18.54% compared to the Original GWO (Linear) value of 0.6050 and significantly outperforms the Exponential Decay variant (Mean: 1.0896). Furthermore, the proposed method exhibited the lowest standard deviation of 0.5713, indicating enhanced stability and robustness in high-dimensional search spaces.

### 2.2. VMD Algorithm

VMD is used to decompose a signal into multiple IMF components by constructing and solving a variational model [26,27]. Each resulting component is characterized by its own center frequency and exhibits a compact distribution in the frequency domain. The core concept of VMD is to achieve adaptive signal decomposition through a mathematical optimization framework, thereby mitigating the end effects and mode mixing issues prevalent in traditional methods, such as EMD [28,29,30].

The specific computational procedure of the VMD algorithm is outlined as follows:

(1) Decompose the signal f(t) into *K* modes $u_{k}(t)$ such that:

(9) $$\sum_{k=1}^{K}u_{k}(t)=f(t)$$

(2) For each IMF component, estimate its center frequency $e^{-j\omega_{K}t}$ and shift the spectrum to the corresponding baseband:

(10) $$\left[\left(\delta (t)+\frac{j}{\pi t}\right)*\mu_{K}(t)\right]e^{-j\omega_{K}t}$$

(3) Minimize the sum of bandwidths of all modes. The variational problem is defined as:

(11) $$\underset{\mu_{K},\omega_{K}}{\min}\left\{\sum_{K}{\left\|\partial_{t}\left[\left(\delta \left(t\right)+\frac{j}{\pi t}\right)*\mu_{K}\left(t\right)\right]e^{-j\omega_{K}t}\right\|}_{2}^{2}\right\}$$

where $\partial_{t}$ denotes the time derivative, $\delta \left(t\right)$ is the Dirac delta function, ∗ represents convolution, and $\omega_{K}$ is the center frequency of the mode. Introduce the Lagrangian multiplier λ(t) and quadratic penalty term α to convert the constrained optimization into an unconstrained problem:

(12) $$\begin{array}{l} L\left(\left\{\mu_{K}\right\},\left\{\omega_{K}\right\},\lambda \right)=\alpha \sum_{K}{\left\|\partial_{t}\left[\left(\delta \left(t\right)+\frac{j}{\pi t}\right)*\mu_{K}\left(t\right)\right]e^{-j\omega_{K}t}\right\|}_{2}^{2}+\\ {\left\|f(t)-\sum_{K}\mu_{K}(t)\right\|}_{2}^{2}+\langle \lambda (t),f(t)-\sum_{K}\mu_{K}(t)\rangle \end{array}$$

The modes, center frequencies, and Lagrangian multiplier are then iteratively updated via frequency-domain operations using the Alternating Direction Method of Multipliers (ADMM).

(4) Terminate iterations when the following condition is met or the maximum cycle count is reached:

(13) $$\sum_{k=1}^{K}\frac{{\left\|u_{k}^{n+1}-u_{k}^{n}\right\|}_{2}^{2}}{{\left\|u_{k}^{n}\right\|}_{2}^{2}}<\epsilon$$

where ϵ is the convergence threshold determining the termination criterion.

### 2.3. NLM Filtering

The fundamental principle of the NLM algorithm operates as follows: for a given target point in a signal, a search neighborhood of size M is defined. Within this neighborhood, the algorithm identifies data points with similar patterns [31,32]. The similarity between each of these points and the target point is quantified, and corresponding weights are assigned accordingly. The denoised value for the target point is then computed as the weighted average of all similar points within the neighborhood, effectively suppressing noise [33,34,35].

Assume the noisy signal is z(i), and the denoised signal after NLM filtering is $\tilde{z}(i)$, such that:

(14) $$\tilde{z}(i)=\frac{1}{M(i)}\sum_{l\in N_{i}}\omega (i,l)z(i)$$

where $M(i)=\sum_{l\in N_{i}}\omega (i,l)$ is the normalization constant; ω(i,l) denotes the weight assigned to z(i), determined by the distance between two points, satisfying 0≤ω(i,l)≤1, and $\sum_{l}\omega (i,l)=1$. The weight is calculated as:

(15) $$\omega (i,l)=\exp\left\{-\frac{\sum_{\delta \in \Delta }\left[z(i+\delta )-z(l+\delta )\right]}{2L_{\Delta }\lambda^{2}}\right\}$$

where λ is the filter parameter, and $L_{\Delta }$ represents the total number of points in the region Δ. Unlike local smoothing filters that average neighboring pixels and often blur structural details, the NLM algorithm suppresses noise while preserving signal transitions by exploiting the repetitive patterns in the time series. Since the weight ω(i,l) is determined by the Gaussian-weighted Euclidean distance between neighborhood patches, only segments with similar motion characteristics contribute to the denoising of the target point. This mechanism ensures that high-frequency physical variations with distinct structural features are assigned higher weights, thereby preventing the excessive suppression of real signal oscillations.

### 2.4. GVMD-NLM Algorithm

The improved GWO optimizes parameters [*K*, *α*] using Permutation Entropy (PE) as the fitness function. In the specific implementation, the calculation of PE [36] relies on the phase space reconstruction of the signal. Based on previous studies and experimental testing, the embedding dimension is set to m=5 and the time delay is set to τ=1. The length of the data segment for entropy calculation aligns with the length of the IMF component. This configuration ensures that the algorithm effectively captures the complexity of the signal structure without excessive computational cost.

**Figure 2:**
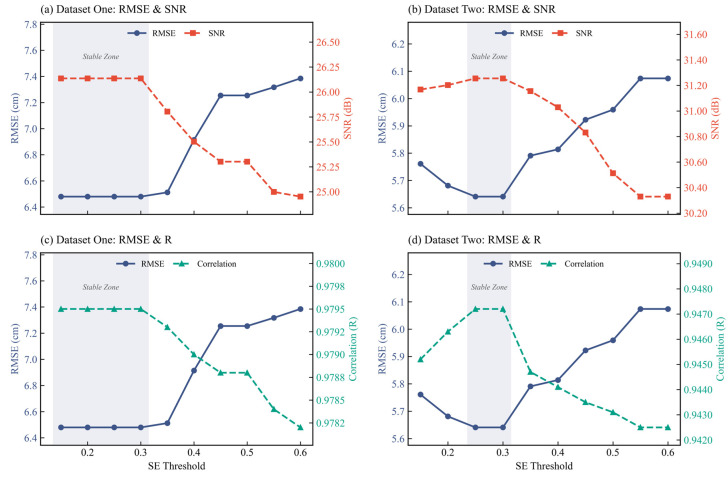
Sensitivity analysis of the SE threshold on RMSE, SNR, and Correlation Coefficient (R) for both datasets: (**a**) RMSE and SNR for Dataset One; (**b**) RMSE and SNR for Dataset Two; (**c**) RMSE and Correlation (R) for Dataset One; (**d**) RMSE and Correlation (R) for Dataset Two.

After determining the optimal parameter combination, the GNSS elevation time series is decomposed into *K* IMF components. Following the methodology in [37], SE is introduced to distinguish between noise-dominant and signal-dominant components. To strictly verify the robustness of the SE threshold and circumvent the limitations of empirical selection, a quantitative sensitivity analysis was conducted. We performed a threshold sweep ranging from 0.15 to 0.60 with a step size of 0.05 on both experimental datasets. The impacts of the threshold variation on the denoising performance metrics, including RMSE, SNR, and R, are illustrated in Figure 2.

As visualized in the figure, a distinct ‘stable zone’ is observed for thresholds between 0.15 and 0.30. Within this interval, the denoising performance remains optimal and strictly stable. For instance, in Dataset One, the RMSE maintains a minimum of 6.48 cm and the SNR stabilizes at 26.14 dB. Similarly, Dataset Two achieves its optimal RMSE of 5.64 cm within the range of 0.25 to 0.30. However, when the threshold exceeds 0.30, a clear inflection point appears followed by a rapid degradation in performance. Specifically, at a threshold of 0.40, the RMSE increases to 6.91 cm for Dataset One and 5.81 cm for Dataset Two. This indicates that higher thresholds cause high-frequency noise components to be erroneously retained as signals. Consequently, 0.30 is identified not only as physically consistent with the 1 Hz sampling rate but also as the statistical upper bound of the optimal performance interval.

**Figure 3:**
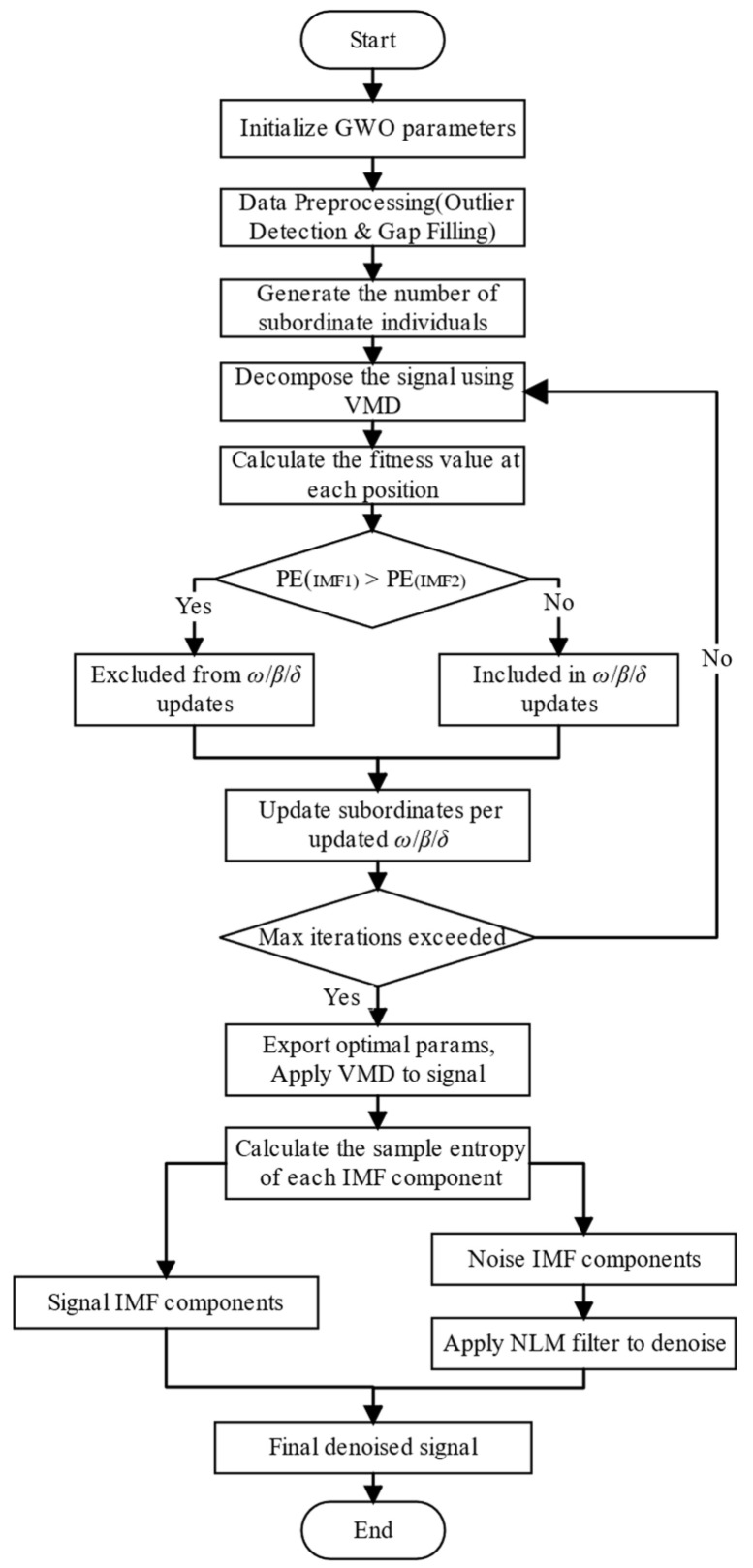
GVMD-NLM denoising workflow.

The optimized VMD algorithm, integrated with sample entropy, is employed to segregate the components into signal and noise categories. The noise components are subsequently processed using NLM filtering to recover any residual effective information. This filtered output is then combined with the signal components to reconstruct the final denoised signal, thereby forming the proposed GVMD-NLM denoising method as illustrated in Figure 3:

(1) Initialize the GWO by defining the search spaces for parameters *K* and *α* as [4, 15] and [500, 3000], respectively. These initialization ranges were determined based on the spectral characteristics of GNSS buoy elevation signals and preliminary trials. The lower bound of *K* ensures basic signal decomposition, while the upper bound prevents mode mixing caused by over-decomposition. Similarly, the range for α covers the typical bandwidth requirements for separating hydrodynamic signals from noise. Sensitivity analyses performed with expanded search ranges (e.g., K∈[2,20],α∈[100,5000]) confirmed that the algorithm consistently converges to the same optimal parameters, demonstrating that the method is robust to the selection of initial search boundaries. Generate an initial population of 30 grey wolves and set the maximum iteration count to 20 [38,39]. To ensure the reproducibility of the optimization results and mitigate the impact of stochastic initialization, a fixed random-seed policy is employed, and the final parameters are determined based on the convergence stability observed over 50 independent runs. Experimental results indicated that the fitness value (Permutation Entropy) typically stabilizes within 20 iterations. Increasing the iteration count beyond 20 significantly increases the computational burden due to the repetitive VMD decomposition without yielding a statistically significant improvement in the denoising performance

(2) Decompose the signal using VMD. A criterion is incorporated to prevent signal over-decomposition:

(16) $$H_{p}(IMF_{1})>H_{p}(IMF_{2})$$

In Equation (16), $H_{p}$ specifically represents the Permutation Entropy of the intrinsic mode function. This inequality serves as an effective criterion for over-decomposition because, in a properly decomposed sequence, the first IMF typically captures the highest frequency noise components, resulting in the highest PE value. If the PE of ${IMF}_{1}$ is significantly greater than ${IMF}_{2}$ beyond a certain threshold, it indicates that the noise has been successfully isolated; conversely, a convergence or reversal of these values suggests that signal components are being leaked into multiple modes, signifying over-decomposition. Grey wolf individuals that satisfy this criterion are excluded from updating the positions of the *β*, *δ*, and *ω* wolves.

(3) Update the positions of all grey wolves according to their fitness values to refine the search direction.

(4) Execute the iterative loop until the termination condition is met, ultimately outputting the optimal parameter combination [*K*, *α*].

(5) Decompose the target signal via VMD using the optimal parameters [*K*, *α*] obtained in Step (4), resulting in *K* IMF components.

(6) Classify the obtained IMF components into signal-dominant and noise-dominant categories based on the established threshold criterion. Specifically, calculate the SE value for each IMF; if SE<0.3, the component is identified as effective signal (representing deterministic biotic/hydrodynamic trends); if SE≥0.3, it is identified as noise-dominant (representing stochastic disturbances) and designated for NLM filtering.

(7) Sum all the noise IMF components to reconstruct a composite noise signal. Apply NLM filtering to this composite noise signal to extract the partial effective information it contains. The NLM filter parameters were determined through systematic sensitivity analysis (detailed in Section 2.5) and were ultimately set as: search window *M* = 100, neighborhood window *p* = 20, and filter parameter *h* = 10.

(8) Reconstruct the denoised signal by combining the signal IMF components obtained in step (6) with the filtered information obtained in step (7).

### 2.5. Sensitivity Analysis of NLM Parameters

To ensure methodological rigor in selecting NLM filter parameters and circumvent the limitations of subjective empirical settings, this study adopted a systematic heuristic method based on sensitivity analysis to determine the search window size (*M*), neighborhood window size (*p*), and filtering parameter (*h*). Using a segment of the GNSS elevation time series data from Experiment One, we varied one parameter at a time while holding the others constant. The Root Mean Square Error (RMSE) and Signal-to-Noise Ratio (SNR) served as evaluation metrics to identify the optimal parameter set. The initial ranges for the NLM parameters were selected based on two considerations. First, commonly used intervals for time series denoising reported in the literature were referenced to ensure comparability [40]. Second, the inherent characteristics of the dataset used in this study—such as sampling rate, noise level, and series length—were considered. Pre-experimental observations helped determine the boundaries of parameter effects, ensuring the tested ranges encompass potential optimal values while avoiding computationally inefficient regions.

**Figure 4:**
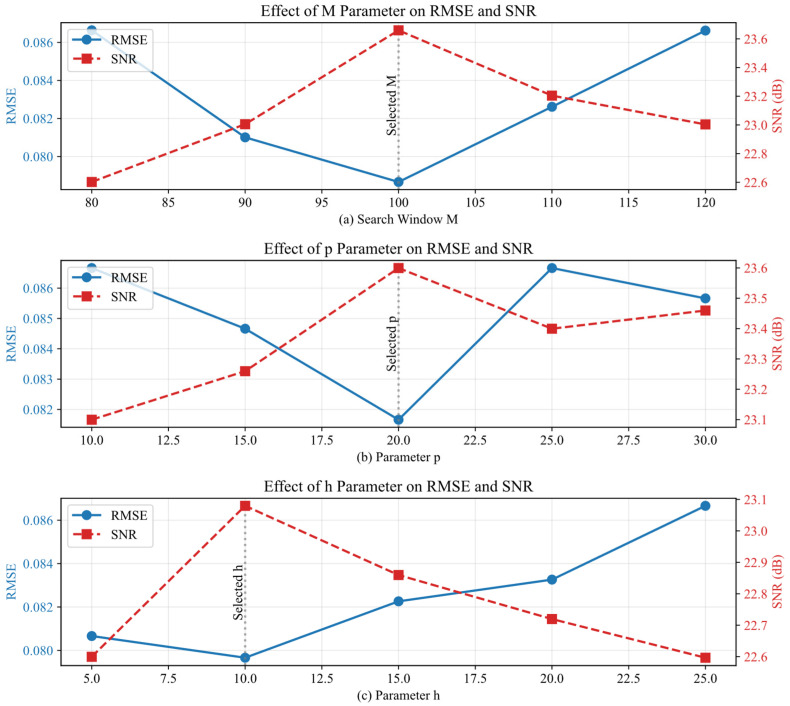
Sensitivity analysis of NLM parameters: (**a**) search window *M*, (**b**) neighborhood window *p*, (**c**) filter parameter *h* on RMSE and SNR.

The specific test ranges are as follows: With *p* = 20 and *h* = 10 fixed, the search window *M* was tested across [80, 90, 100, 110, 120]. This range was determined through preliminary experiments: when *M* < 80, denoising was insufficient; when *M* > 120, performance gains plateaued while computational cost increased substantially. Thus, this interval was chosen to balance denoising effectiveness and efficiency. With *M* = 100 and *h* = 10 fixed, the neighborhood window *p* was varied within [10, 15, 20, 25, 30]. The value of *p* is typically set much smaller than *M* to preserve the validity of local similarity comparisons. The selected range covers common neighborhood sizes cited in the literature and maintains a reasonable proportion relative to *M* = 100. With *M* = 100 and *p* = 20 fixed, the filtering parameter *h* was varied across [5, 10, 15, 20, 25]. As *h* governs the degree of smoothing and is highly sensitive to the outcome, this range was designed to cover the full spectrum from under-smoothing to over-smoothing. The experimental results are shown in Figure 4.

Figure 4a illustrates that as the search window size *M* increases from 80 to 100, the RMSE decreases significantly to a minimum of 0.084, while the SNR rises to a peak of 23.2 dB. Theoretically, expanding *M* increases the search domain Δ in Equation (15), allowing the algorithm to identify more redundant structural patterns globally. This enriches the candidate set for the weighted averaging in Equation (14), thereby reducing the variance of the estimator. However, when *M* exceeds 100, the probability of including heterogeneous structures increases. According to Equation (15), if unrelated patches are included within Δ, their Euclidean distances may still accidentally result in non-negligible weights due to noise interference, thereby introducing bias into the reconstruction. Consequently, *M* = 100 was selected to achieve an optimal balance between denoising performance and computational efficiency. As shown in Figure 4b, the neighborhood window *p* achieves optimal performance at 20. The parameter *p* determines the dimensionality of the similarity vectors used in the Euclidean distance calculation in Equation (15). A *p* value that is too small renders the distance metric sensitive to random noise spikes, leading to inaccurate weight distribution. Conversely, an excessively large *p* implies a stricter similarity criterion, which, while robust, reduces the distinctiveness of local features and causes the blurring of signal edges during the weighted averaging process. Analysis of Figure 4c indicates that the filtering parameter *h* is highly sensitive to the denoising outcome. The RMSE reaches its minimum (0.084) and the SNR is 22.8 dB at *h* = 10. The parameter *h* acts as the decay control in the exponential function of Equation (15). Mathematically, it regulates the bandwidth of the similarity kernel. When *h* is set too low (*h* < 10), the exponential term decays rapidly, assigning significant weights only to patches that are nearly identical, which results in insufficient noise reduction (under-smoothing). Conversely, a large *h* (*h* > 10) flattens the weight distribution, causing dissimilar patches to contribute disproportionately to the weighted average in Equation (14), leading to the loss of high-frequency signal details (over-smoothing). Thus, *h* = 10 was identified as the optimal value.

Based on the comprehensive sensitivity analysis, the final parameter combination for the NLM filter was determined as *M* = 100, *p* = 20, and *h* = 10. This selection is grounded in an objective comparison of performance metrics, which ensures an optimized denoising effect and establishes a reliable parameter configuration for subsequent experimental stages.

## 3. Results and Discussion

### 3.1. Study Area and Experimental Data

The study area is located at the Dongguan Waterway Wharf in Shilong Town, Dongguan City, a crucial node within the Pearl River Delta’s intricate coastal waterway network. This site was selected for data collection due to its representative and dynamic hydrodynamic characteristics. Unlike open ocean environments, this coastal waterway is subject to complex interactions between tidal currents, river discharge, and anthropogenic disturbances such as vessel wakes, making it an ideal proving ground for evaluating the robustness of GNSS denoising algorithms. Its geographical position offers the advantage of experiencing significant water level fluctuations driven by tides, while being logistically accessible for the deployment and maintenance of the GNSS buoy.

**Figure 5:**
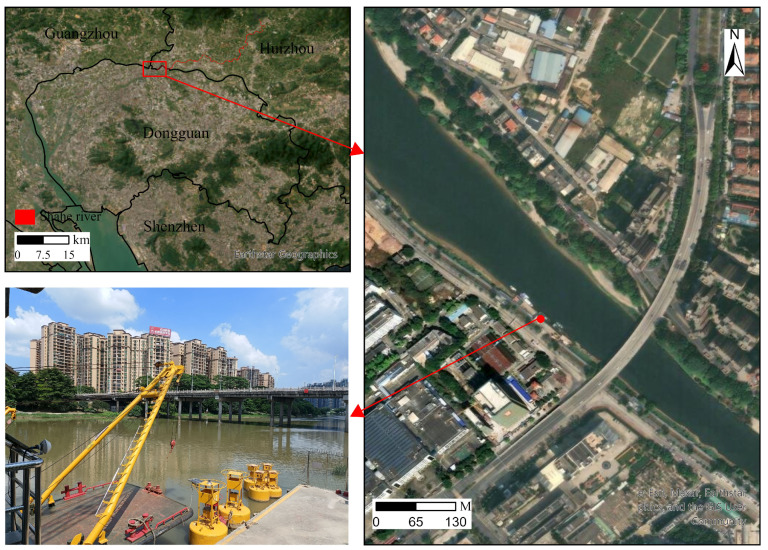
Study area illustration: (**top-left**) overview of Shilong Town and the Sha River, (**right**) top view of Dongguan Waterway Wharf, (**bottom-left**) real-scene photo of the wharf.

Figure 5 illustrates the study area, which consists of three parts: the top-left panel shows an overview of Shilong Town and the Sha River where the GNSS buoy was deployed, the right panel presents a top view of the Dongguan Waterway Wharf, and the bottom-left panel displays a real-scene photo of the wharf.

Two datasets of GNSS buoy elevation time series were collected for denoising experiments in this challenging coastal waterway environment. Dataset One was recorded for one hour at a sampling frequency of 1 Hz. Dataset Two was recorded for one day at the same sampling frequency. Both datasets were obtained using a GNSS buoy (Yantai Navigation Lights Electronic Technology Co., Ltd., Yantai, China) deployed at the wharf, providing real-time elevation coordinates in a coastal waterway.

To ensure the reproducibility of the measurement system, the specific hardware and environmental configurations are detailed as follows. The buoy system is equipped with a Quectel LG290P positioning module (Hefei Yunlu Information Technology Co., Ltd., Hefei, China) and a high-precision external GNSS antenna Type HXS508404TS-W70B (Shenzhen Hongxin IoT Technology Co., Ltd., Shenzhen, China). Structurally, the buoy relies on a pontoon for buoyancy and is secured by an anchor chain at the bottom. It operates on an independent power supply system consisting of solar panels and storage batteries, with position monitoring and navigation warning functions achieved through the integrated positioning antenna and a beacon light. The GNSS positioning was conducted using the Qianxun CORS (Continuously Operating Reference Stations) network mode. Regarding environmental noise, the study area is primarily affected by multipath effects, signal occlusion, and dynamic disturbances. Specifically, specular reflections from the water surface are the core cause of low-frequency multipath errors in the elevation time series. Non-line-of-sight (NLOS) propagation caused by riparian vegetation severely degrades the satellite geometric configuration (particularly the Vertical Dilution of Precision, VDOP). Furthermore, high-frequency random shaking induced by waves and electromagnetic radiation from internal equipment introduce complex non-stationary noise superimposed on the observations.

Prior to applying the denoising algorithm, the raw elevation time series underwent a rigorous preprocessing phase to ensure data quality and continuity. This procedure consisted of two key steps: (1) Outlier Removal: The 3σ criterion was utilized to identify and eliminate gross errors caused by multipath effects or receiver instability. (2) Gap Filling: To satisfy the strict continuity requirement of the VMD algorithm, the Regularized Expectation Maximization (RegEM) algorithm was employed to interpolate missing epochs. Unlike traditional linear interpolation, RegEM is a data-driven approach that leverages the covariance structure of the signal, thereby preserving the hydrodynamic characteristics of the original data while reconstructing gaps.

### 3.2. Experimental Evaluation Metrics

To comprehensively evaluate the denoising performance of the proposed GVMD-NLM method, this study conducts two experiments using field-measured data. The effectiveness of the proposed method is validated through rigorous comparisons with SSA, CEEMDAN, standard VMD, and the optimized GWO-VMD approach [41]. During the evaluation, the *RMSE*, *SNR*, and correlation coefficient (*R*) are adopted as performance metrics [42], which are defined as follows:

(17) $$RMSE=\sqrt{\frac{1}{N}\sum_{i=1}^{N}{\left(x_{i}-y_{i}\right)}^{2}}$$

(18) $$SNR=10\mathrm{lg}\left(\frac{\sum_{i=1}^{N}x_{i}^{2}}{\sum_{i=1}^{N}{\left(x_{i}-y_{i}\right)}^{2}}\right)$$

(19) $$R(x,y)=\frac{\sum_{i=1}^{N}(x_{i}-\bar{x})(y_{i}-\bar{y})}{\sqrt{\sum_{i=1}^{N}{\left(x_{i}-\bar{x}\right)}^{2}}\sqrt{\sum_{i=1}^{N}{\left(y_{i}-\bar{y}\right)}^{2}}}$$

where i denotes the time index of the coordinate epoch; $\bar{x}$ and $\bar{y}$ are the mean values of x and y respectively. x represents the denoised signal; y represents the ‘true’ water level signal. In this study, to ensure rigorous validation, the independent water level observations from a co-located tide gauge station at the Dongguan Waterway Wharf were utilized as the ground truth y. Prior to the calculation, the tide gauge data were temporally synchronized with the GNSS buoy observations, and the systematic constant bias between the GNSS ellipsoidal height and the tide gauge vertical datum was removed.

### 3.3. Denoising Analysis of Dataset One

To validate the effectiveness and applicability of the proposed GVMD-NLM method in denoising measured GNSS elevation time series, elevation coordinate data collected by a GNSS buoy deployed at the Dongguan Waterway Wharf (Shilong Town, Dongguan City) were utilized. The GNSS buoy was operated at a sampling frequency of 1 Hz for a duration of one hour.

**Figure 6:**
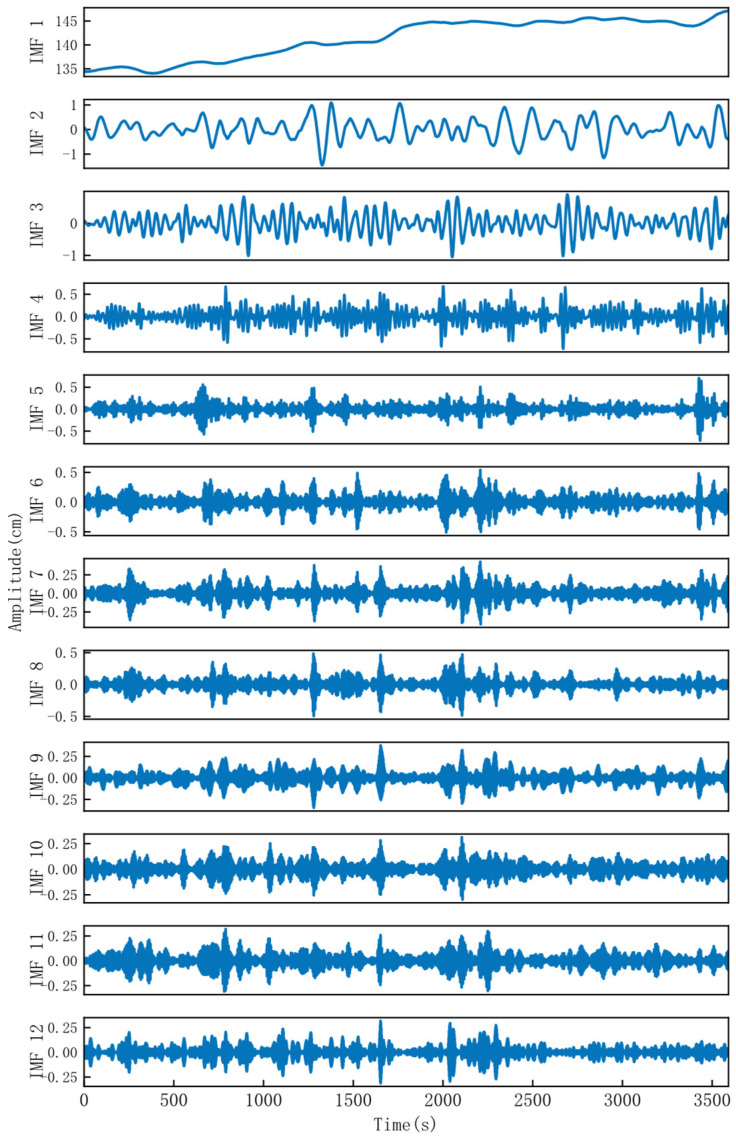
Results of VMD decomposition.

**Figure 7:**
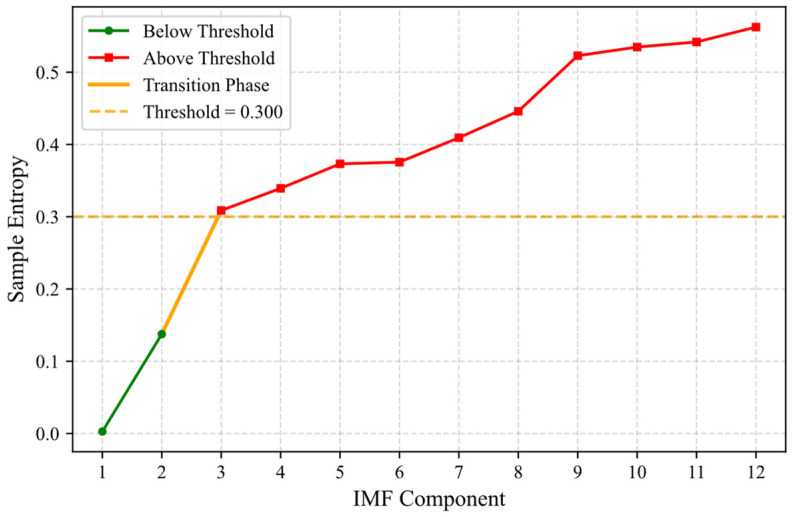
SE values of each IMF component.

The proposed GVMD-NLM method was applied to the dataset with the implementation parameters rigorously configured as described in Section 2. Specifically, the Permutation Entropy calculation used an embedding dimension of 5 with a delay of 1, and the NLM filter utilized the optimized parameter set (*M* = 100, *p* = 20, *h* = 10) derived from the sensitivity analysis. The optimized VMD decomposition yielded the parameter combination *K* = 12 and *α* = 1553, as illustrated in Figure 6. It can be observed that IMF1 aligns closely with the original signal, capturing the overall slow-varying trend of the elevation. IMF2 and IMF3 exhibit increased amplitudes relative to IMF1, along with gradually rising frequencies, and demonstrate relatively clear periodic fluctuations, which may correspond to certain medium-period influencing factors. In contrast, IMF4 to IMF12 show significantly larger amplitudes and are characterized by high-frequency oscillations, likely associated with short-term, high-frequency variations embedded in the elevation time series. The SE values for each IMF component were computed, with the results summarized in Figure 7 and Table 2.

**Figure 8:**
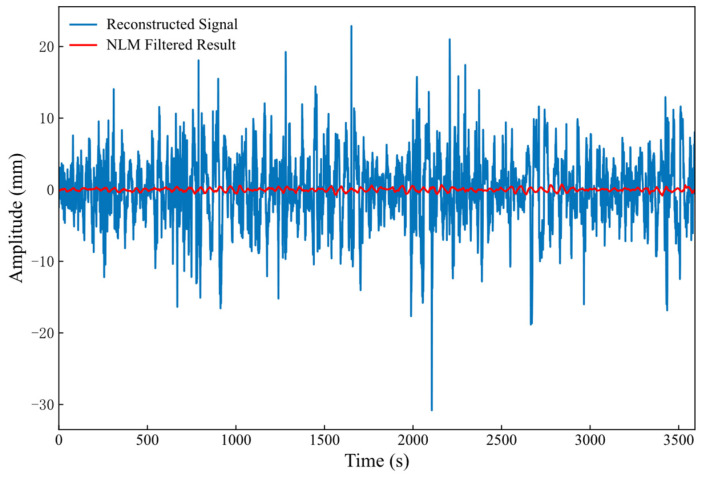
Effect of NLM filtering on Dataset One.

A careful analysis of the data presented in Table 2 indicates that the SE values of the first two IMF components are both below the predetermined threshold of 0.3. Consequently, these two components were identified as signal-dominant, extracted, and reconstructed to form the effective signal component. The remaining ten components were summed and reconstructed to form a composite noise component, which was then processed using NLM filtering. The detailed outcomes of this procedure are shown in Figure 8. Regarding the visual smoothness of the GVMD-NLM curves, it is essential to distinguish between effective noise suppression and potential information loss. While the reconstructed noise components exhibit considerable volatility, oscillating frequently between −30 mm and 20 mm, the filtered signal presents a notably smoother trajectory that successfully retains the fundamental physical trends of the original data. This “smoothness” primarily reflects the removal of stochastic measurement errors rather than the attenuation of actual hydrodynamic signals, which is quantitatively supported by the high correlation coefficient of 0.9798 for Dataset One. Such fidelity is further guaranteed by the Sample Entropy based selection mechanism, which ensures that low-frequency IMFs containing the primary physical energy remain unprocessed by the NLM filter, while the NLM algorithm itself preserves structural features through its non-local weighting mechanism.

**Figure 9:**
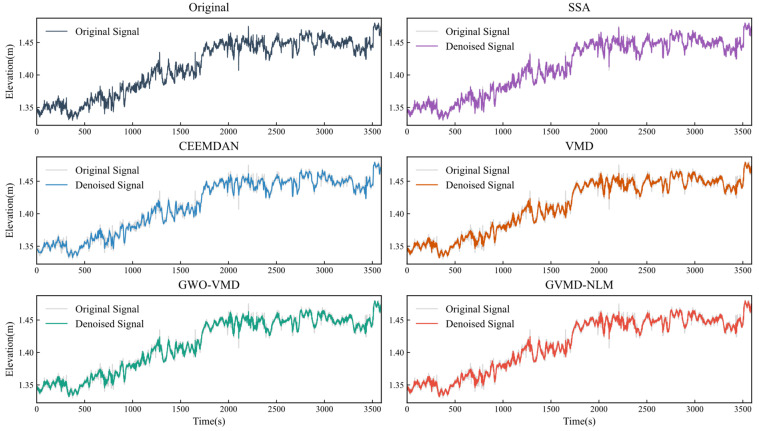
Comparison of original signal and denoised signals in Dataset One.

To comprehensively verify the advanced nature and robustness of the proposed method, the GVMD-NLM algorithm was evaluated against four well-established comparative baselines: Singular Spectrum Analysis (SSA), Complete Ensemble Empirical Mode Decomposition with Adaptive Noise (CEEMDAN), standard VMD, and GWO-VMD. Regarding the parameter settings for these methods: Based on the spectral characteristics of the experimental data and literature recommendations [11,16], the window length L for SSA was set to 500 for Dataset One and 3600 for Dataset Two. For CEEMDAN, to balance reconstruction error and computational efficiency, the amplitude of the added white noise was set to 0.2, and the number of ensemble trials was set to 100. For the standard VMD implementation, the parameter set [K,α]=[12,2000] was determined through multiple experimental trials, while the GWO-VMD method yielded an optimal parameter combination of [K,α]=[12,1553]. The denoising results and corresponding performance metrics for all five methods are presented in Figure 9 and Table 3. Analysis of the results indicates that while standard VMD and SSA achieve a fundamental level of noise reduction, their performance in smoothing fine-scale details is limited. The CEEMDAN and GWO-VMD methods demonstrate improved adaptability and noise suppression capabilities. Notably, among all comparative approaches, the GVMD-NLM method generates the smoothest denoised signal with noise appearing almost completely suppressed. Furthermore, it maintains excellent preservation of detailed signal characteristics. Quantitatively, as detailed in Table 3, the proposed GVMD-NLM method clearly demonstrates significantly superior denoising effectiveness compared to the other four methods.

In this experiment, the GVMD-NLM method demonstrated superior performance over four comparative baselines, as evidenced by its reduced RMSE and enhanced SNR and R values. This superiority confirms its efficacy in denoising GNSS elevation time series. The method incorporates an improved GWO algorithm that achieves adaptive optimization of critical VMD parameters. By employing a nonlinear convergence factor adjustment mechanism, the algorithm strengthens global search capability, overcoming the limitations of empirical parameter selection. Furthermore, the integration of a sample entropy-based component screening mechanism allows for effective discrimination between signal and noise. The subsequent application of NLM filtering to the identified high-frequency noise components enables substantial noise suppression while preserving local details. Quantitative results further substantiate this superiority, showing respective reductions of 26.04%, 17.87%, 24.28%, and 13.47% in RMSE, increments of 0.61%, 0.27%, 0.31%, and 0.14% in the R, and improvements of 11.13%, 7.00%, 10.18%, and 5.05% in SNR compared to the SSA, CEEMDAN, VMD, and GWO-VMD methods.

### 3.4. Denoising Analysis of Dataset Two

**Figure 10:**
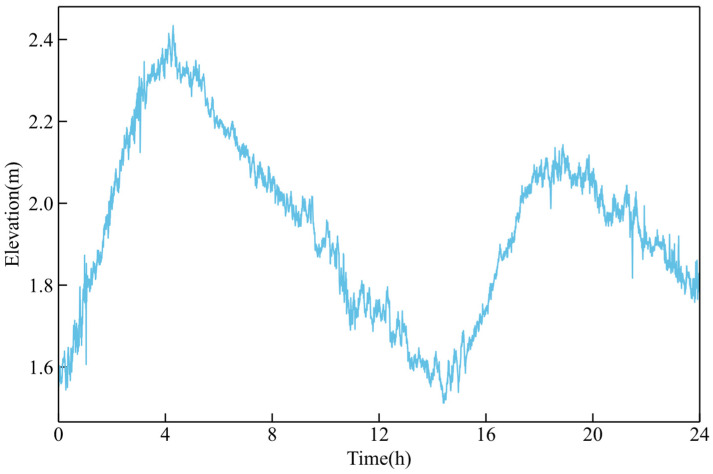
Waveform of the measured elevation time series.

To further assess the effectiveness of the GVMD-NLM method, GNSS buoy data comprising a one-day elevation time series sampled at 1 Hz was selected for additional validation. The profile of the original elevation time series is presented in Figure 10.

The signal was decomposed using the optimized VMD, resulting in parameters *K* = 14 and *α* = 900. Based on the SE threshold criterion, IMFs 1 to 3 (as listed in Table 4) were identified as effective signal components. The remaining IMFs, whose SE values exceeded the predetermined threshold, were selected and reconstructed into a composite noise component. This procedure successfully achieved a clear separation and effective extraction of the noise embedded within the original signal.

**Figure 11:**
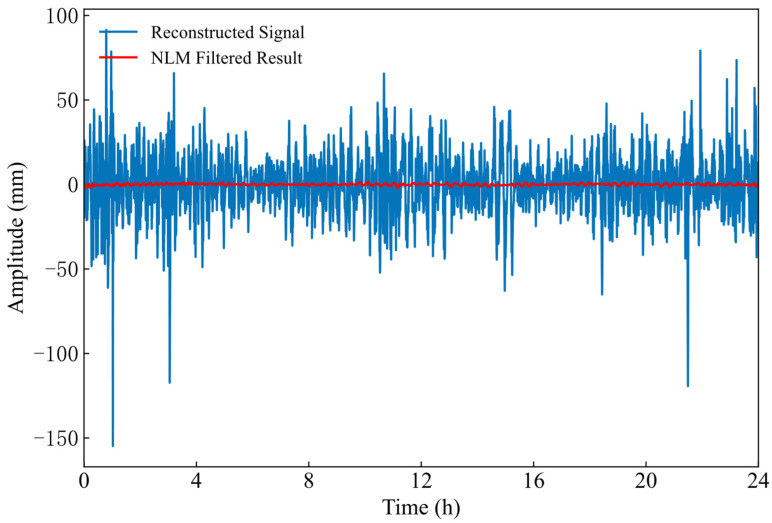
Effect of NLM filtering on Dataset Two.

The first three IMF components were extracted and reconstructed to form the effective signal component. The successful application of the identical threshold to this dataset, despite its differing duration and hydrological dynamics compared to Dataset One, empirically validates the robustness of the selected criterion. Crucially, the decision to include the third IMF component in the signal reconstruction was strictly driven by the SE criterion. As detailed in Table 4, the SE value of IMF3 in this dataset is 0.226, which falls below the established threshold of 0.3. This contrasts with Dataset One, where the third component exceeded the threshold (SE = 0.309) and was consequently classified as noise. This distinction highlights the adaptability of the proposed method: it dynamically determines the number of effective signal components based solely on their entropy characteristics, without relying on a fixed, pre-determined count. To verify the robustness and transferability of the selected NLM parameters, the same parameter set determined in Section 2.5 was directly applied to Dataset Two without further tuning. Subsequently, the remaining eleven components were similarly reconstructed into a composite noise component, which then underwent NLM filtering using these fixed parameters. The results of this processing are presented in Figure 11. Analysis of the figure indicates that applying NLM filtering to the noise component effectively recovers detailed signal information that was previously obscured by noise.

**Figure 12:**
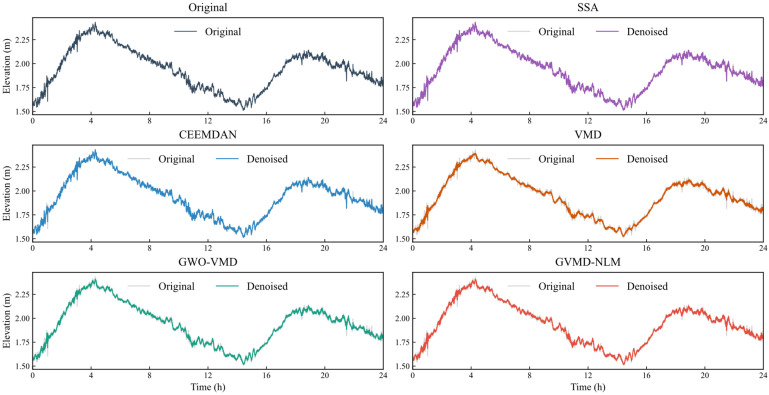
Comparison of original signal and denoised signals in Dataset Two.

The comparative results with SSA, CEEMDAN, standard VMD, and GWO-VMD are presented in Figure 12 and Table 5. While advanced decomposition methods like CEEMDAN and GWO-VMD demonstrate efficacy in suppressing most noise components, ensuring high accuracy in extracting nonlinear signals, they do not fully address complex signal characteristics, often resulting in suboptimal preservation of subtle authentic information. Similarly, although SSA and standard VMD produce relatively smooth denoised signals, they tend to retain substantial high-frequency noise or over-smooth details, leading to inferior performance. Compared to these four methods, the GVMD-NLM approach exhibits significant advantages. The denoised signal obtained through GVMD-NLM shows a high degree of conformity with the original data, underscoring its pronounced efficacy. It more accurately preserves the essential features and critical information of the original signal, ensuring both authenticity and reliability. Quantitatively, the method achieves reductions in RMSE of 28.87%, 17.12%, 18.45%, and 10.26%, increases in the correlation coefficient (R) of 4.04%, 1.77%, 1.81%, and 0.83%, and improvements in SNR of 10.48%, 5.52%, 6.02%, and 3.11%, respectively, relative to the SSA, CEEMDAN, VMD, and GWO-VMD methods. Although formal statistical hypothesis testing was not conducted, the consistent superiority of the GVMD-NLM method across two independent datasets with distinct durations and varying environmental conditions demonstrates the empirical robustness of the results. The substantial margins of improvement in RMSE observed in both cases further indicate that these performance gains are reliable and consistent, rather than attributable to random variations.

### 3.5. Frequency Domain Validation

To rigorously verify that the proposed GVMD-NLM method preserves physically meaningful high-frequency components and to address potential concerns regarding over-smoothing, a frequency-domain analysis was conducted. Taking Dataset One as a representative experimental case, we performed a Power Spectral Density (PSD) comparison and a band-energy retention analysis.

**Figure 13:**
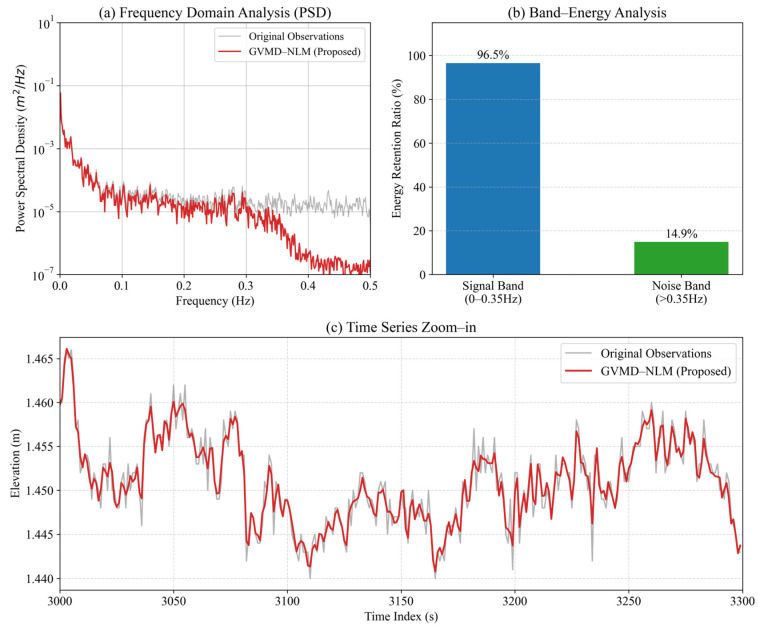
Frequency domain validation and signal preservation assessment. (**a**) Comparison of Power Spectral Density (PSD) between original and denoised signals; (**b**) Quantitative band-energy retention analysis; (**c**) Zoomed-in view showing the preservation of waveform details.

As illustrated in Figure 13a, the PSD of the GVMD-NLM denoised signal exhibits a high degree of consistency with the original observations in the low-to-medium frequency range (0–0.35 Hz). This frequency band captures dominant hydrodynamic forces, specifically superimposing tidal variations and wind-driven waves (typically 0.1–0.3 Hz). While acknowledging that PSD is a global metric and does not strictly guarantee every local micro-variation, the strong spectral overlap confirms that the algorithm effectively preserves the macroscopic energy of these physical processes without evident attenuation. Conversely, in the high-frequency band (>0.35 Hz), the PSD of the denoised signal shows a significant drop compared to the original signal, indicating the effective suppression of stochastic noise and measurement jitter.

To quantify this performance, a Band-Energy Analysis was performed, as shown in Figure 13b. The energy retention ratio is calculated for the signal-dominant band and the noise-dominant band, respectively. The results show that the GVMD-NLM method retains 96.5% of the energy in the signal band, confirming that the main physical information is intact. Meanwhile, only 14.9% of the energy is retained in the noise band, proving that the majority of high-frequency interference has been eliminated.

Furthermore, the time series zoom-in view in Figure 13c corroborates the spectral analysis. The denoised signal (red line) closely tracks the local peaks and troughs of the original data, capturing the detailed fluctuations rather than simply generating a smooth mean curve. This confirms that the GVMD-NLM method achieves a balance between noise reduction and feature preservation, avoiding the over-smoothing issue common in conventional filtering.

## 4. Conclusions

This paper presents a GVMD-NLM algorithm for denoising GNSS buoy elevation coordinate time series, which is optimized by an improved grey wolf optimizer. Compared to established baselines such as SSA and CEEMDAN as well as conventional VMD-based approaches, the proposed method addresses the limitations of traditional VMD that relies on empirical parameter presetting. It enhances decomposition accuracy and adaptability through parameter optimization, while effectively suppressing noise and better preserving detailed features in GNSS coordinate time series signals. Furthermore, the method successfully mitigates the distortion typically occurring at signal endpoints in traditional approaches, thereby significantly improving the completeness and reliability of denoising results. Experimental validation through two separate experiments using measured data confirms that the proposed method achieves superior denoising performance against all comparative methods in both cases.

Despite the promising results, this study identifies avenues for future research to address current limitations. First, regarding operational deployment, future work will focus on code optimization to reduce computational latency for real-time application in GNSS buoy systems. Second, while this study utilized specific field datasets, we plan to extend validation to longer-term and multi-site GNSS data to rigorously assess the method’s robustness across varying geographic and environmental conditions. Third, we will explore the integration of advanced data-driven techniques, such as machine learning or deep learning, to further enhance the adaptability of parameter optimization and signal decomposition.

Finally, comprehensive simulation experiments will be conducted to evaluate performance under specific noise types (e.g., impulsive or correlated noise) and extreme non-stationary scenarios, ensuring reliability under diverse monitoring conditions.

## Figures and Tables

**Table 1:** Quantitative performance evaluation of the three GWO variants.

Method	Final	Mean	Std
Original GWO (Linear)	0.5974	0.6050	0.6125
Exponential GWO	1.2063	1.0896	0.6947
Proposed Sigmoid-GWO	0.4552	0.4928	0.5713

**Table 2:** Statistics of SE values for each IMF component in Dataset One.

Component	SE Value	Component	SE Value
${IMF}_{1}$	0.00276051	${IMF}_{7}$	0.40933166
${IMF}_{2}$	0.13754923	${IMF}_{8}$	0.44591853
${IMF}_{3}$	0.30865377	${IMF}_{9}$	0.52281935
${IMF}_{4}$	0.33927505	${IMF}_{10}$	0.53475674
${IMF}_{5}$	0.37320051	${IMF}_{11}$	0.54183353
${IMF}_{6}$	0.37551942	${IMF}_{12}$	0.56240406

**Table 3:** Denoising performance of the five methods in Dataset One.

Denoising Method	RMSE/cm	R	SNR/dB
SSA	8.7318	0.9739	23.5341
CEEMDAN	7.8637	0.9772	24.4435
VMD	8.5295	0.9768	23.7377
GWO-VMD	7.4636	0.9784	24.8972
GVMD-NLM	6.4583	0.9798	26.1539

**Table 4:** Statistics of SE values for each IMF component in Dataset Two.

Component	SE Value	Component	SE Value
${IMF}_{1}$	0.0078193	${IMF}_{8}$	0.44261666
${IMF}_{2}$	0.17475437	${IMF}_{9}$	0.4465918
${IMF}_{3}$	0.22558907	${IMF}_{10}$	0.4639434
${IMF}_{4}$	0.32478298	${IMF}_{11}$	0.51301881
${IMF}_{5}$	0.34468286	${IMF}_{12}$	0.51930041
${IMF}_{6}$	0.36174541	${IMF}_{13}$	0.54450293
${IMF}_{7}$	0.36600569	${IMF}_{14}$	0.54763879

**Table 5:** Denoising performance of the five methods in Dataset Two.

Denoising Method	RMSE/cm	R	SNR/dB
SSA	7.7577	0.9128	28.2360
CEEMDAN	6.6582	0.9331	29.5636
VMD	6.7670	0.9329	29.4228
GWO-VMD	6.1495	0.9419	30.2540
GVMD-NLM	5.5184	0.9498	31.1945

## Data Availability

The raw data supporting the conclusions of this article will be made available by the authors on request.
